# Improving oral health of children in four Balkan countries: A qualitative study among health professionals

**DOI:** 10.3389/froh.2022.1068384

**Published:** 2023-01-09

**Authors:** Enes Karamehmedovic, Poul Erik Petersen, Maren L. Agdal, Jorma I. Virtanen

**Affiliations:** ^1^ Oral Health Centre of Expertise, Stavanger, Norway; ^2^ Department of Clinical Dentistry, University of Bergen, Bergen, Norway; ^3^ Institute of Odontology, University of Copenhagen, Copenhagen, Denmark; ^4^ Oral Health Centre of Expertise in Western Norway, Bergen, Norway; ^5^ Institute of Dentistry, University of Turku, Turku, Finland

**Keywords:** children, health policy, fluorides, oral health prevention, oral health promotion

## Abstract

Data from the WHO show that the oral health situation for children in the Balkan region of Europe is poor. This study aims to identify obstacles that prevent the implementation of an effective oral health program for children in the Balkan area. The study investigates the challenges that people encounter in increasing awareness, attending to dental care, and complying with examinations.

**Methods:**

This qualitative study targeted experts in preventive dentistry and oral health promotion in four countries in the Balkan region. Purposive sampling was used to recruit the participants. Data were collected in 2021 using individual in-depth interviews with participants from Albania, Bosnia and Herzegovina, Croatia, and Serbia. The study applied the thematic analysis method.

**Results:**

The experts reported four main challenges that hindered the implementation of a prevention program and regular patient attendance: (1) lack of knowledge, (2) the exclusion of oral health from overall health, (3) organization of services, and (4) skepticism of fluoride. The participants identified knowledge gaps among the general population, dental staff, and other health professionals regarding the prevention of oral diseases.

**Conclusion:**

The findings of this study may be used to promote and improve oral health among children in the identified areas and to benefit people in the region and elsewhere. This study sheds light on the existing barriers in a region where people lack information.

## Introduction

Oral diseases are chronic and progressive in nature and affect people beginning in childhood and continuing through adolescence and adulthood (1). Poor dental health and untreated oral diseases cause persistent and severe pain and malnutrition in children and negatively affect their growth and school performance, thereby escalating social inequalities (1–3). Although oral health is generally neglected, it is essential for an individual's quality of life (4). The World Health Organization (WHO) considers various oral conditions, such as dental caries, periodontal disease, tooth loss, oral cancer, HIV/AIDS oral lesions, and oro-dental trauma, as severe public health problems^1^. Health programs generally overlook oral diseases, although being immensely prevalent and severe public health problems (5). In addition, oral diseases cause a significant burden and economic cost to healthcare systems (1, 6), surpassing the expenses for treating cancer, heart disease, stroke, and dementia (7). Oral diseases are influenced by poor oral hygiene habits and frequent among people with high sugar consumption and users of tobacco. Currently, oral diseases are escalating in middle- and low-income countries and recur more in the lowest socioeconomic groups of all countries (1, 8, 9). Petersen and Kwan (10) explained the importance of reducing inequalities in oral health and based on WHO's strategies for noncommunicable disease prevention, published new approaches for oral disease prevention, and health promotion.

In 2015, the WHO published a policy for disease prevention through reducing sugar consumption (11), and its Oral Health Program launched strategies for controlling sugar consumption (12). The WHO has demonstrated that manufactured foods high in sugar are a preferred source of energy in southern European countries (13). Because foods with high sugar content are affordable to people with economic challenges, they contribute to widening the gap in oral health standards for people from different socioeconomic backgrounds. The WHO's data show that the oral health situation for children in the Balkan region in Europe is poor (6, 14). For example, in Albania, Bosnia and Herzegovina, and Serbia, less than 50% of 5- and 6-year-olds are caries-free. Although no data are available for Croatia, children in these four countries have a higher risk of tooth decay than the regional average (14, 15). Unlike other European countries, these countries have hardly had a long-term, population-wide preventive program that is adequately designed and implemented. Instead, efforts have focused primarily on individual and behavioral aspects.

Over the past two decades, the oral health systems of the Balkan countries have changed significantly. The burden of dental care has moved from public to private dentistry, with a greater focus on advanced technology and treatment. The rationale for this change and the effect of the privatization of dental services on oral health in the Balkan countries remain unclear. Furthermore, none of the countries has wholly adopted a full-scale fluoridation system. As part of the WHO's public health strategies to prevent tooth decay, it has advocated for the provision of sufficient fluoride intake in areas that do not have it (16). This can be achieved through specific measures, such as the topical application of fluoride gels or fluoride supplements (17), oral hygiene instructions with fluoride toothpaste (18), or other inexpensive measures, including drinking water, salt, or milk fluoridation (16).

Research on tackling the poor oral health situation for children in the Balkan region is scarce (19). This study aims to identify the obstacles that prevent the implementation of an effective oral health program, reduce the existing knowledge gap, and address the underlying causative factors of poor oral health. It is also essential to investigate the challenges and resistance that people encounter so that actions can be taken to foster a broader interest in performing daily preventive measures and to increase attendance to dental care.

## Material and methods

### Study design and data collection

We applied an exploratory qualitative study design (20) and adhered to the Consolidated Criteria for Reporting Qualitative Research (COREQ) (21). We collected data by conducting individual in-depth interviews with participants from four Balkan countries: Albania, Bosnia and Herzegovina, Croatia, and Serbia. The first author conducted interviews between August and December 2021. Before the interviews, the interviewer received an in-depth training to encourage the interviewees to openly present their views (22).

### Participants and participant selection

The following criteria were established for participant selection: (1) specialization in pedodontics or preventive dentistry, (2) experience with health promotion and oral disease prevention programs, and (3) affiliation with a dental university or a governmental/public health office. An email invitation was sent to all potential candidates, and the selection yielded nine participants, including seven women and two men. All the participants were employed, except for one expert who had retired. Before the study, the participants were familiarized with the interviewers' reasons and interest in this research. After conducting a preliminary analysis of the interviews, a high level of agreement was reached, and no new themes emerged. It was concluded that the research question was answered, and that the theoretical saturation needed for the qualitative research was reached (20).

### Data collection

Interviews were conducted to gain insight into the personal perceptions of dental healthcare professionals, preventive dentistry specialists, and members of governmental/public health offices. We used purposive sampling and pre-established inclusion criteria to obtain a credible group of experts (23). We created a semistructured interview guide with a series of open-ended questions to ensure uniformity in data collection throughout the interviews, while allowing for a flexible and natural flow of conversation to stimulate thoughts and reflection (20). The interviewer asked the participants questions about promoting oral health and preventing oral diseases among youth from their countries. We sought information about current programs or upcoming plans to improve oral health and to identify the challenges and barriers to improving oral health at the national level. Additionally, the interviewer asked the experts about their views on manageable and sustainable ways of increasing fluoride exposure and reducing the intake of free sugars.

Due to COVID travel restrictions, the interviews were conducted using Zoom Video Communications software (Zoom Video Communications Inc., 2016). All the participants spoke their native language, except for the Albanian experts with whom the interviews were conducted in English. All the interviews were conducted by the first author (EK), who is fluent in all the languages used. The interviews lasted 40–74 min and were recorded and transcribed verbatim. The same author later translated the interviews into English, and then an independent dental professional back-translated them to the original language to ensure correct translations. All identifiable information was omitted during the translation of the interviews.

### Ethical considerations

The Regional Ethical Committee for Medical Research concluded that the committee did not need to consider this project because patients were not involved in the study (Case number: 286069). In compliance with international standards for ethics in research involving human subjects, all participants were informed about the study's aims and purposes, the anonymity and confidentiality of participants' responses, and their right to withdraw their data from the study at any point without negative consequences. Furthermore, a written consent form was provided to each participant prior to the interviews.

### Data analysis

The verbatim records of the interviews resulted in raw data material of 67 typed pages. No interviews were repeated. The thematic analysis adhered to the principles of Braun and Clarke (20), which provide an accessible and theoretically flexible approach to analyzing qualitative data. A computerized program for textual analysis (NVivo 12.6.0 Qualitative Data Analysis Software Version 2020) was used to identify and cluster codes from the interviews. The analysis was conducted as follows:

(1) The first author transcribed all interview recordings verbatim.
(2) Two of the authors (EK and MLA) individually differentiated and labeled the segments of each text with a code.
(3) The codes were categorized into a code tree, and two authors compared them to identify four dominant themes.
(4) The fourth author (JIV) then inspected and confirmed the themes based on the code tree.

## Results

During the analysis, a code tree consisting of 27 codes was developed to improve coding efficiency and streamline the research process. Four main thematic categories were obtained and conceptualized: (1) lack of knowledge, (2) the exclusion of oral health from overall health, (3) organization of services, and (4) skepticism toward fluoride.

### Lack of knowledge

The data showed a consensus among the participants that the population in the identified countries lacked knowledge about personal preventive measures to reduce dental caries. The following quote from one of the participants demonstrates the lack of knowledge about daily toothbrushing:

*The problem here is that people don't brush their teeth. So, I would rather base everything on “brush, brush, brush.” People tell me, “I bought my child an electric toothbrush,” but it doesn't make a difference; it's not better than a normal toothbrush if you don't use it, you know.* (p. 8)

In addition to the lack of knowledge about essential oral disease prevention, the general population in the four countries was ignorant of the importance of early and regular preventive dental visits.

The participants found the situation to be a vicious circle in which people fail to seek dental care before their children are in pain. This makes the practitioner preoccupied with treating caries instead of educating parents on daily preventive oral healthcare measures. The following quote from a participant addresses this vicious cycle:

*Education about the importance of oral hygiene, the importance of first control at the early stages, regular check-ups. First, you must explain the importance of brushing their teeth. You can't explain it to [parents] when they don't bring [the children]. There is no prevention [in public clinics], so [you have] even more urgent patients, which leaves less time to do prevention.* (p. 3)

In addition, the society at large lacks knowledge about the reasons for caries and preventive measures. According to the participants, the population in this region claims that the current knowledge is experience-based and is passed from older to younger generations. However, that knowledge may contain misinformation, which makes it difficult for people to accept new information or practices. One participant stated that “We keep doing everything we can, but there is no point. I think it's the mentality of the people” (p. 3). Another participant explained “The parents close their ears, and that's just it, there's nothing you can do there. You explain it to them, but yes, in one ear and out the other. They say that bad teeth run in their family” (p. 5).

Another participant expressed a similar sentiment and stated the following:

*So, in our population, there is that question “Why would a child go see a dentist, it doesn't have its teeth yet,” and it's not that you need to create a habit, how are you going to feed a child, night feeding, breastfeeding at night, those are all things that people don't understand, about it and they know little about.* (p. 4)

The tradition is to treat and fix dental issues rather than to prevent them. Therefore, to change the attitudes and knowledge of the general population, education must target people from a young age, continue through adulthood, and focus on the elderly.

The analysis of the interviews indicated that the lack of knowledge and need for education are multilevel. The participants expressed that pediatricians caring for newborn babies and their mothers are unaware of their crucial preventive function in improving oral health and health behaviors from a child's early age. The following quote exemplifies the participants' concerns: “A pediatrician should be able to send a child [to a dentist] … and emphasize the importance [of good oral health] to the parents” (p. 6).

However, the interviews indicated that a deficiency in education makes medical doctors underprepared for this important behavioral-focused task. One participant stated “Doctors don't understand [what to say] … they try, but they do not have dental classes in their curriculum; how could they understand [?]” (p. 9).

Another participant stated, “Pediatricians and family physicians, maybe they don't have [as] much knowledge about oral health as they should, so they're not promoting oral health well enough among children and parents” (p. 4).

According to the participants, dental professionals who are uneducated about prevention do not emphasize preventive care. Resolving dental problems is a far more common practice than acting to prevent them. Education about preventive care should be a more significant part of the dental education curriculum. Dental students prefer caries treatment and other dental treatment procedures. The following quote explains the lack of interest in preventive dental care:

*We used to have a class on the importance of good oral hygiene and maintaining oral health in the first year of university, and they even canceled that a couple of years back. It is important to start with those things early because later, when [students] come to year four or something, and they taste blood in oral surgery or something, it is difficult to bring them back to prevention.* (p. 8)

Negative consequences arise from the lack of preventive education. Children develop early childhood caries and are not taken to a dentist before their caries worsen. One participant shared her experience: “Starting from children themselves, we can see that the child visits for the first time a dentist when [he or she] is starting school or because of the pain, then it is actually, already too late” (p. 4).

### The exclusion of oral health from overall health

The participants expressed a general concern that oral health is not perceived as part of the population's general health in their countries. The fundamental concept that oral health differs from general health leads to a common misunderstanding that oral health should be restored rather than cared for and maintained. When people do not consider oral health as part of general health, they fail to comply with oral hygiene and regular dental attendance, which are needed for disease prevention. Regarding this issue, one participant said, “We see dentistry outside health care; this is a big mistake; I disagree. I was one of the people who said that dentistry, we are separated from medicine, it's not a good idea, if we're going to work in prevention, we should work together” (p. 1). Another participant stated “I always say that oral health is a part of [overall health], the mouth is just a part of the body, it's about the health of everything, the overall health of the person” (p. 2).

According to the participants, general health and oral health should be regarded as connected and inclusive, as they share the same risk factors. However, the participants pointed to the existing distinction between general health and oral health, starting from an institutional level and continuing throughout all aspects of society. The participants believed that this distinction could be mitigated by fundamentally changing the idea of health and cooperating on common health goals. A participant stated “We should work together with medicine, so dentistry and medicine or other health professions [work] together through the common risk factors” (p. 1). Another participant explained “It is a challenge that society finally realizes the importance of prevention and keeping good oral health” (p. 8).

The participants appealed for cooperation between the private and public dental sectors to better emphasize oral disease prevention. Preserving oral health as the first step toward overall health was one of the participants' distinctive points of view.

One participant stated, “Prevention is the only way how to keep a good oral health, not just good oral health but general health” (p. 4).

Another participant explained, “Private clinics are more focused on the treatment than the prevention because of the profit. We should try to support prevention with insurance or financing schemes. Let's say by starting to cover some preventive procedures by the government or something” (p. 1).

### Organization of dental services and preventive dental care

According to the participants, oral health services have shifted from public to private clinics. Governments have reduced funding for public clinics, leading to disparities in facilities and staffing and reducing confidence in the public sector. One participant stated “There was closing and cutting budgets on the public section, that's why we don't have this much of public section” (p. 2). Another participant said “I think that the parents lost, as everything is moving toward privatization, they have lost faith in public dentistry” (p. 7).

In addition, the participants highlighted the increased economic gains of private clinics as the change in infrastructure has outpaced public clinics. One participant explained the following:

*But there is a huge discrepancy, and there has been a massive privatization of dental services. It*'s *the same, and all these Balkan countries are similar regarding this stuff. The same thinking, so it leads to the same problems. Now, we have a lot of socially vulnerable people here, unfortunately, but that dentistry in public offices is not as good as in private because they lack materials. It is also influenced by those funds granted by the state, and the management always chooses the cheapest option, which turns out to be the most expensive one in the end. In that sense, public clinics can't compete with private clinics.* (p. 9)

From the participants' perspectives, the change to privatization leads to dental health services that focus less on preventive dental care because it generates a lower income compared with that from dental treatment. One participant stated the following about this situation:

*I am talking openly here; it's not just the patient, we, or most of us as professionals, are not motivated, we are focused on seminars and congresses for points, and people are only focused on that which brings money in dentistry, so implants and prosthodontics.* (p. 8)

The participants all agreed that national institutes and public dentistry lack effort and interest in prevention. Oral disease prevention programs have been nonexistent or mostly implemented through individual efforts. The lack of collaboration across countries has led to poor planning and thereby poor execution of the programs. One participant stated “Well, we don't have a national program as far as I know. Prevention is more connected to smaller areas or regions where you have your plan and program and some goals that public clinics must reach” (p. 7).

The informants thought that health institutions should become more involved and work together to develop and implement a broader preventive program. For the program to be beneficial, it should have initial guidelines, ongoing assessments, and specific targets that must be met while reaching the population at large. As one participant explained:

*Everyone should be included in this process, not just dentists, but public health offices, the ministry of health, everyone should be involved. It's not just the concern of some small group of people, but a concern of a large group of people, and it's about health.* (p. 2)

Another participant stated:

*The profession's responsibility is to create a good system that can be controlled and evaluated so it's not just written on paper and not conducted as it should be. Later evaluation is when you check the results that have been accomplished because you must start every program with research to analyze the situation. After a couple of years, depending on how long the program lasts, if the average caries indices or gingival disease indices are not being reduced, then that program didn't yield results, and we must search for the cause of why it didn't work.* (p. 8)

In three of the identified countries, public dental care was free for people until the age of 18. Several participants thought it was unfortunate that free public dental health service was mistaken for service of a lower quality because it does not have a cost. When using free dentistry, people do not realize the cost of poor dental health and dental treatment. Therefore, it was suggested that an itemized bill should be presented to the parents to demonstrate the actual cost of the service provided. A participant stated, “I don't think it would be bad that someone, at least something symbolic, that they receive some bill [that shows] how much a service costs, so they realize and see how much they would have to pay if that wasn't free” (p. 6).

The poor oral health situation results from the culmination of the systemic disregard for oral health, which has been escalating for years. As one participant described “You're left with an unimproved consciousness about oral health across multiple generations. I think this complete story for the last 30 years, and general carelessness about oral health, is finally breeding results” (p. 9).

### Skepticism about fluorides

The general uncertainty and skepticism toward fluorides by the population at large was the final theme that emerged as a barrier to good oral health. While some participants believed that skepticism was present in the general population, others expressed personal concerns about dentists who are skeptical about the use of fluorides. A participant stated “I'm a little bit skeptic about using added fluorides, not only to foods but also taking fluorides like it used to be a long time ago, tablets of fluoride. Because of the systematic effects of it” (p. 2). Another participant explained “There is this misconception about the usage of systemic fluorides, even among dentists, for babies and small children. Even among pediatricians, if they should suggest or not use fluoride products” (p. 3).

The uncertainty about fluorides seemed to be founded on inadequate knowledge about its advantages and disadvantages when consumed or administered topically. The participants expressed the need for the promotion of knowledge to achieve better preventive dental care practices. One participant stated the following:

*There is still a huge debate regarding [the] plusses and minuses regarding fluorides. Because there were both endogenous and exogenic use of fluorides, stories were going around if it is cancerous or not, and as soon as they hear some negative news, people back out.* (p. 9)

The participants thought that the misconception that fluoride, regardless of its form or concentration, always has adverse effects emerged because of information about its potential harmful effects when consumed in large amounts. When patients mistrust the safety of fluorides and question their preventive effects, they avoid using fluorides, and this has detrimental effects on dental health by causing severe dental decay.

One participant explained how this misconception has led to mistrust in the advice from dental professionals and WHO recommendations:

*But what about those that have three or four active caries lesions, and they don't want to use fluoride toothpaste because they read somewhere on the internet that it is bad and they see those signs like fluoride with skeleton heads, you know those danger signs, and then they come to our clinic and talk about it and try to convince us that it is bad and dangerous. We try to explain that it is a therapeutic medium and why it is necessary. However, in this modern internet age, they interpret it in their own way and try to avoid that medium to maintain oral hygiene.* (p. 6)

The participants suggested that toothpaste with fluorides should be subsidized because toothpaste without fluorides is often less expensive and may be preferred despite the dentist's recommendations. However, the participants claimed that regardless of their opinions on fluorides, the public at large still disregards guidelines for daily toothbrushing. Poor oral hygiene, which excludes fluorides, is regarded as one of the most significant barriers to oral health for the countries in this study. One participant explained the following:

*We have toothpaste for children up to the age of six without fluorides at all. They are also cheaper than those with fluoride, so of course, people will buy them. But they buy [them], and they do not even brush, or brush rarely.* (p. 3)

The participants recommended that evidence and accurate information be provided through social media to alter the public's perception of fluorides and overall oral health and to increase knowledge about fluorides. A participant stated “The evidence and information should be given to the people to support this fluoridation” (p. 1). Another participant explained “So, through media, we should use the instruments of social marketing, which is very important, where you put your emphasis and your power so someone could have a healthy lifestyle and they care for their oral and overall health” (p. 9).

## Discussion

The study participants observed a lack of knowledge about oral health in the general population of the Balkan countries. People do not consider oral health to be a part of overall health, and the fundamental understanding of the organization of various governmental services is incomplete. The public's mistrust of fluorides is a key factor preventing the employment of an effective oral health promotion program in the region. The participants were frustrated and felt powerless to do their work in promoting oral health because laypeople preferred to trust what had been passed down from others rather than adopting expert opinions. Moreover, the Balkan experts reported that dental practitioners and other health professionals who do not sufficiently prioritize prevention have knowledge gaps that must be addressed. This is consistent with previous studies revealing knowledge gaps among dental staff (24) and other health professionals (25) regarding the prevention of oral diseases. Effective oral health promotion strategies require knowledge and competence in all aspects of health (1). Appropriate education and training are crucial for health professionals to successfully address health inequities (26).

### Integration of work for oral health

The health professionals who participated in the study mentioned the isolation of oral health from general health as another challenge. Furthermore, the participants emphasized that because general health and oral health share the same risk factors, they should be considered using an interrelated and comprehensive approach. The WHO has recommended incorporating oral health into general health in national noncommunicable disease prevention programs and has encouraged the use of the “Common Risk Factor” approach (27). National administrators widely accept this approach in dental public health (14). The World Health Assembly introduced this approach in the “2007 Resolution on Oral Health: Action Plan for Promotion and Integrated Disease Prevention” (28) and the World Health Resolution on Oral Health endorsed the strategy in 2021 (29). The two policy statements explain the responsibility of countries in integrating appropriate oral disease prevention and health promotion into public health actions. Moreover, activities must encompass the interaction between oral health and general health, and the approach should tackle the underlying social determinants of health (29). During the past several years, the WHO has emphasized that school health education is a crucial element of health promotion, which seeks to improve health knowledge and develop healthy lifestyles (30). Educational interventions integrated with health services development are essential for advancing health systems. These actions increase the likelihood of sustainability and constant support and positively affect oral health behaviors in the long term (31).

### Health policies

According to the participants, the distinction between general health and oral health starts at the institutional level and extends throughout all facets of society. As a result of the combined efforts of governmental organizations and agencies with sufficient economic resources, healthcare systems have become central to improving and preserving the public's health (14). However, significant inequities exist between low- and middle-income countries and high-income countries in the delivery of preventive care for all population groups (5, 32, 33). This can be attributed to several differences in the health systems, such as the absence of oral health policies, an inadequate workforce to implement prevention measures, insufficient financial support, and little encouragement from dentists in the application of prevention because of the incentives of restorative care (14).

Similar to most countries in Central and Eastern Europe, the Balkan countries lack organized prevention programs. At the same time, dental care has shifted from public to private services in recent years (34). This change may have improved individual patient therapy due to technology, materials, and infrastructure developments (34). However, population-wide protocols that are evidence-based and cost-effective are missing in improving daily care and encouraging people's perspectives on oral health (1, 34, 35). This privatized model of dentistry is also inappropriate for managing the disease at the population level, as it neither addresses the existing burden of disease nor delivers effective prevention (1) but is primarily driven by profit and consumerism (35).

The burden of childhood dental caries remains high in countries around the world (36). In most Eastern European countries, a significant proportion of carious lesions are untreated because of inadequacy, unaffordability, or absence of oral healthcare services, which results in poor overall health and quality of life (9, 34). The participants also identified this as one of the main concerns of this study. Collaboration between the private and public dental sectors is essential to ensure that greater emphasis is placed on oral disease prevention. With affordable population-wide programs, dental caries is mainly preventable, whereas treatment is costly and unavailable in many low- and middle-income countries (5, 11).

During the last 6 years, Croatia has implemented a nationwide program called “Dental Passport” to provide comprehensive preventive dental examinations for children aged 6–12 years (37). Although the program is in its early stages, it has shown an increase in regular examinations (37). However, the literature indicates that such downstream approaches to behavioral actions tend to result in short-term, positive changes in oral health behaviors, with problems commencing in the long-term improvement of clinical outcomes (32, 35). Furthermore, such downstream approaches have been demonstrated to exacerbate health inequalities among various socioeconomic groups, as privileged individuals often acquire more benefits (32, 33).

### Fluorides

The findings of this study highlight a mistrust in the effectiveness of fluorides, resulting in inadequate exposure and creating a significant barrier to the improvement of oral health. Fluoridation programs are effective in preventing dental caries (38), and the WHO recommends fluoride administration for dental caries prevention through water, milk, and salt (39). Evidence of water fluoridation has been made in Ireland, and positive effects of salt fluoridation have been shown in Switzerland (40). In addition, the practical outcome of school-based milk fluoridation has been documented in Bulgaria (40), and the benefit of using fluoridated toothpaste among schoolchildren has been confirmed in Slovenia (41).

In summary, a decline in the prevalence of caries can be traced to the decreased consumption of dietary free sugars (3, 13), systematic school-based preventive programs (14), and improved oral hygiene practices that incorporate toothpastes containing fluorides (18), fluoride mouth rinses, or topical fluorides (17). The WHO has stated that dental caries can be prevented by avoiding dietary free sugars (11). Consequently, Petersen highlighted that decreasing sugar consumption should be prioritized in population-directed oral disease prevention strategies (42). Previous studies have shown a significant association between the amount of sugar consumed and the number of caries lesions (3, 11, 13).

Importantly, upstream interventions that make the population aware of and motivated to achieve better oral health are necessary to address the problem successfully (26). National governments and policymakers should initiate upstream interventions within the population to tackle broader social determinants (e.g., education, harmful health behavior, and sugar consumption) (32, 35). The best approach to prevent major oral diseases and decrease the cost of dental services for individuals and society is to adopt healthy behaviors (31), such as daily toothbrushing (18), regular exposure to fluoride sources (16, 17), and limited sugar consumption (3, 13).

### Strengths and limitations of the study

Qualitative research methodology is used to understand the experiences and attitudes of patients and the proficiencies of the community or healthcare professionals. This study uncovers existing challenges in a region where information is deficient (19). The interviews were conducted online because of the COVID pandemic. Although face-to-face interviews are generally preferred, the busy experts thought it was feasible to discuss the issues in their own environments and welcomed the online approach. The verbatim records of the interviews resulted in a vast amount of data, which were analyzed using appropriate thematic methods. The purposive sample was at the lower end of the range employed in thematic analysis (20). However, a high degree of consensus appeared in the interviews. According to the current literature, a sample size of 8–12 people is considered suitable (43).

## Conclusions

This study sheds light on the existing barriers related to oral health promotion and disease prevention in a region where information is deficient. The findings may be used to promote and improve oral health for children in the identified areas and benefit people of the countries in the Balkan region and elsewhere.

## Data Availability

The raw data supporting the conclusions of this article will be made available by the authors, without undue reservation.

## Footnotes

^1^ https://www.who.int/health-topics/oral-health

## References

[1] Peres MA Macpherson LMD Weyant RJ Daly B Venturelli R Mathur MR Oral diseases: a global public health challenge. Lancet. (2019) 394(10194):249–60. 10.1016/S0140-6736(19)31146-8 31327369

[2] Karimy M Higgs P Abadi SS Armoon B Araban M Rouhani MR Oral health behavior among school children aged 11-13 years in Saveh, Iran: an evaluation of a theory-driven intervention. BMC Pediatr. (2020) 20(1):476. 10.1186/s12887-020-02381-6 33050893 PMC7552527

[3] Mansur EKM. Primary prevention of dental caries: an overview. Int J Clin Prev Dent. (2020) 16(4):143–8. 10.15236/ijcpd.2020.16.4.143

[4] Glick M Williams DM Kleinman DV Vujicic M Watt RG Weyant RJ. A new definition for oral health developed by the FDI World Dental Federation opens the door to a universal definition of oral health. Am J Orthod Dentofacial Orthop. (2017) 151(2):229–31. 10.1016/j.ajodo.2016.11.010 28153139

[5] Benzian H Hobdell M Holmgren C Yee R Monse B Barnard JT Political priority of global oral health: an analysis of reasons for international neglect: political priority of global oral health. Int Dent J. (2011) 61(3):124–30. 10.1111/j.1875-595X.2011.00028.x 21692782 PMC9374826

[6] Petersen PE. Percentage of children aged 6 years (or 5) who are free of dental caries in WHO Europe. Who.int. (2018). Available at: https://www.who.int/news-room/fact-sheets/detail/oral-health (Accessed September 2022).

[7] Petersen PE. The world oral health report 2003: continuous improvement of oral health in the 21st century—the approach of the WHO global oral health programme. Community Dent Oral Epidemiol. (2003) 31(s1):3–24. 10.1046/j.2003.com122.x 15015736

[8] Garcia I Tabak LA. Global oral health inequalities: the view from a research funder: the view from a research funder. Adv Dent Res. (2011) 23(2):207–10. 10.1177/0022034511402015 21490232 PMC3144034

[9] Susarla SM Trimble M Sokal-Gutierrez K. Cross-sectional analysis of oral healthcare vs. general healthcare utilization in five low- and middle-income countries. Front Oral Health. (2022) 3:911110. 10.3389/froh.2022.911110 35815119 PMC9259954

[10] Petersen PE Kwan S. Equity, social determinants and public health programmes—the case of oral health: equity, social determinants and public health programmes. Community Dent Oral Epidemiol. (2011) 39(6):481–7. 10.1111/j.1600-0528.2011.00623.x 21623864

[11] World Health Organization (WHO). Guideline: sugars intake for adults and children. Genève: World Health Organization (2015). 25905159

[12] Moynihan P Makino Y Petersen PE Ogawa H. Implications of WHO guideline on sugars for dental health professionals. Community Dent Oral Epidemiol. (2018) 46(1):1–7. 10.1111/cdoe.12353 29168887

[13] World Health Organization, Regional Office for Europe. Incentives and disincentives for reducing sugar in manufactured foods: an exploratory supply chain analysis: a set of insights for member states in the context of the WHO European food and nutrition action plan 2015–2020. Europe: World Health Organization. Regional Office for Europe (2017).

[14] Petersen PE Baez RJ Ogawa H. Global application of oral disease prevention and health promotion as measured 10 years after the 2007 world health assembly statement on oral health. Community Dent Oral Epidemiol. (2020) 48(4):338–48. 10.1111/cdoe.12538 32383537 PMC7496398

[15] Silveira Moreira R. Epidemiology of dental caries in the world. In: Virdi MS, editor. Oral health care—pediatric, research, epidemiology and clinical practices. London: InTech (2012) 8:149–68.

[16] World Health Organization. Preventing disease through healthy environments: inadequate or excess fluoride: a major public health concern. World Health Organization (2019). https://apps.who.int/iris/handle/10665/329484. License: CC BY-NC-SA 3.0 IGO

[17] Sicca C Bobbio E Quartuccio N Nicolò G Cistaro A. Prevention of dental caries: a review of effective treatments. J Clin Exp Dent. (2016) 8(5):e604–10. 10.4317/jced.52890 27957278 PMC5149099

[18] Walsh T Worthington HV Glenny A-M Appelbe P Marinho VC Shi X. Fluoride toothpastes of different concentrations for preventing dental caries in children and adolescents. Cochrane Database Syst Rev. (2010) 20(1):1–173: CD007868. 10.1002/14651858.CD007868.pub2 20091655

[19] Karamehmedovic E Bajric E Virtanen JI. Oral health behaviour of nine-year-old children and their parents in Sarajevo. Int J Environ Res Public Health. (2021) 18:3235. 10.3390/ijerph18063235 33800979 PMC8003837

[20] Braun V Clarke V. Using thematic analysis in psychology. Qual Res Psychol. (2006) 3(2):77–101. 10.1191/1478088706qp063oa

[21] Tong A Sainsbury P Craig J. Consolidated criteria for reporting qualitative research (COREQ): a 32-item checklist for interviews and focus groups. Int J Qual Health Care. (2007) 19(6):349–57. 10.1093/intqhc/mzm042 17872937

[22] Dicicco-Bloom B Crabtree BF. The qualitative research interview. Med Educ. (2006) 40(4):314–21. 10.1111/j.1365-2929.2006.02418.x 16573666

[23] Tongco MDC. Purposive sampling as a tool for informant selection. Ethnobot Res Appl. (2007) 5:147. 10.17348/era.5.0.147-158

[24] Threlfall AG Milsom KM Hunt CM Tickle M Blinkhorn AS. Exploring the content of the advice provided by general dental practitioners to help prevent caries in young children. Br Dent J. (2007) 202(3):E9. 10.1038/bdj.2007.46 17256013

[25] Richards W Filipponi T Roberts-Burt V. Mind the gap! A comparison of oral health knowledge between dental, healthcare professionals and the public. Br Dent J. (2014) 216(4):E7. 10.1038/sj.bdj.2014.100 24557409

[26] Allen M Allen J Hogarth S Marmot M. Working for health equity: the role of health professionals. Institute of Health Equity. Available at: https://www.instituteofhealthequity.org/resources-reports/working-for-health-equity-the-role-of-health-professionals (Accessed March 2022).

[27] Sheiham A Watt RG. The common risk factor approach: a rational basis for promoting oral health: common risk factor approach. Community Dent Oral Epidemiol. (2000) 28(6):399–406. 10.1034/j.1600-0528.2000.028006399.x 11106011

[28] World Health Organization (WHO). Oral health: action plan for promotion and integrated disease prevention (2007). https://apps.who.int/iris/handle/10665/21909

[29] World Health Organization. Seventy-fourth world health assembly (2021). Available at: https://www.who.int/about/governance/world-health-assembly/seventy-fourth-world-health-assembly (Accessed September 2022).

[30] Jürgensen N Petersen PE. Promoting oral health of children through schools—results from a WHO global survey 2012. Community Dent Health. (2013) 30(4):204–18. 10.1922/CDH_3283Petersen15 24575523

[31] Menegaz AM Silva AER Cascaes AM. Intervenções educativas em serviços de saúde e saúde bucal: revisão sistemática. Rev Saude Publica. (2018) 52:52. 10.11606/s1518-8787.2018052000109 29791531 PMC5953546

[32] Dawson ER Stennett M Daly B Macpherson LMD Cannon P Watt RG. Upstream interventions to promote oral health and reduce socioeconomic oral health inequalities: a scoping review protocol. BMJ Open. (2022) 12(6):e059441. 10.1136/bmjopen-2021-059441 35738648 PMC9226867

[33] Lorenc T Petticrew M Welch V Tugwell P. What types of interventions generate inequalities? Evidence from systematic reviews. J Epidemiol Community Health. (2013) 67(2):190–3. 10.1136/jech-2012-201257 22875078

[34] Petersen PE. Oral health systems in Europe—an overview. Stomatology Edu J. (2022) 9(1):1–5. Available at: https://www.stomaeduj.com/oral-health-systems-in-europe-an-overview/ (Accessed September 2022).

[35] Williams DM Mossey PA Mathur MR. Leadership in global oral health. J Dent. (2019) 87:49–54. 10.1016/j.jdent.2019.05.008 31075367

[36] World Health Organization. Factsheet on oral health and sugars intake. (2019). Available at: https://www.euro.who.int/__data/assets/pdf_file/0009/365850/oral-health-2018-eng.pdf (Accessed September 2022).

[37] Pavić Šimetin I Radić Vuleta M Jurić H Kvesić Jurišić A Malenica A. Program for dental health advancement in children “dental passport”. Acta Stomatol Croat. (2020) 54(2):121–9. 10.15644/asc54/2/1 32801370 PMC7362737

[38] O’Mullane DM Baez RJ Jones S Lennon MA Petersen PE Rugg-Gunn AJ Fluoride and oral health. Community Dent Health. (2016) 33(2):69–99. 10.1922/CDH_3707O'Mullane31 27352462

[39] World Health Organization Regional Office for Europe. Fact sheet on oral health—fluoride and oral health. Copenhagen: WHO Europe (2020).

[40] Petersen PE Kwan S Ogawa H. Long-term evaluation of the clinical effectiveness of community milk fluoridation in Bulgaria. Community Dent Health. (2015) 32(4):199–203. 10.1922/CDH_3629Petersen05 26738215

[41] Vrbič V Vrbič M Petersen PE. Epidemiology of dental caries and disease prevention among 12-year-olds in Slovenia over thirty years (1987-2017). Oral Health Prev Dent. (2020) 18(1):185–96. 10.3290/j.ohpd.a44309 32238991 PMC11654562

[42] Petersen PE. Strengthening of oral health systems: oral health through primary health care. Med Princ Pract. (2014) 23(S1):3–9. 10.1159/000356937 24525450 PMC5586948

[43] Polit DF Beck CT. Essentials of nursing research: appraising evidence for nursing practice. 8th ed. Baltimore, MD: Wolters Kluwer Health/ Lippincott Williams & Wilkins (2014).

